# Satellitome Analysis and Transposable Elements Comparison in Geographically Distant Populations of *Spodoptera frugiperda*

**DOI:** 10.3390/life12040521

**Published:** 2022-03-31

**Authors:** Inzamam Ul Haq, Majid Muhammad, Huang Yuan, Shahbaz Ali, Asim Abbasi, Muhammad Asad, Hafiza Javaria Ashraf, Aroosa Khurshid, Kexin Zhang, Qiangyan Zhang, Changzhong Liu

**Affiliations:** 1 College of Plant Protection, Gansu Agricultural University, No. 1 Yingmen Village, Anning District, Lanzhou 730070, China; inzamam@st.gsau.edu.cn (I.U.H.); aroosakhurshid3@gmail.com (A.K.); 1120425163@qq.com (K.Z.); zhangqiangyan2@163.com (Q.Z.); 2 College of Life Sciences, Shaanxi Normal University, Xi’an 710100, China; majidento07@snnu.edu.cn (M.M.); yuanh@snnu.edu.cn (H.Y.); 3 Department of Agricultural Engineering, Khwaja Fareed University of Engineering and Information Technology, Rahim Yar Khan 64200, Pakistan; shahbaz@kfueit.edu.pk; 4 Department of Zoology, Bahawalpur Campus, University of Central Punjab, Bahawalpur 63100, Pakistan; asimuaf95@gmail.com; 5 College of Life Science, Fujian Agriculture and Forestry University, Fuzhou 350002, China; axadch@fafu.edu.cn; 6 College of Plant Protection, Fujian Agriculture and Forestry University, Fuzhou 350002, China; hafizajavaria@yahoo.com

**Keywords:** *Spodoptera frugiperda*, satellitome analysis, transposable elements, repeat profiling, geographic impact

## Abstract

*Spodoptera frugiperda* (fall armyworm) is a member of the superfamily Noctuoidea that accounts for more than a third of all Lepidoptera and includes a considerable number of agricultural and forest pest species. *Spodoptera frugiperda* is a polyphagous species that is a significant agricultural pest worldwide, emphasizing its economic importance. *Spodoptera frugiperda’s* genome size, assembly, phylogenetic classification, and transcriptome analysis have all been previously described. However, the different studies reported different compositions of repeated DNA sequences that occupied the whole assembled genome, and the *Spodoptera frugiperda* genome also lacks the comprehensive study of dynamic satellite DNA. We conducted a comparative analysis of repetitive DNA across geographically distant populations of *Spodoptera frugiperda*, particularly satellite DNA, using publicly accessible raw genome data from eight different geographical regions. Our results showed that most transposable elements (TEs) were commonly shared across all geographically distant samples, except for the Maverick and PIF/Harbinger elements, which have divergent repeat copies. The TEs age analysis revealed that most TEs families consist of young copies 1–15 million years old; however, PIF/Harbinger has some older/degenerated copies of 30–35 million years old. A total of seven satellite DNA families were discovered, accounting for approximately 0.65% of the entire genome of the *Spodoptera frugiperda* fall armyworm. The repeat profiling analysis of satellite DNA families revealed differential read depth coverage or copy numbers. The satellite DNA families range in size from the lowest 108 bp SfrSat06-108 families to the largest (1824 bp) SfrSat07-1824 family. We did not observe a statistically significant correlation between monomer length and K2P divergence, copy number, or abundance of each satellite family. Our findings suggest that the satellite DNA families identified in *Spodoptera frugiperda* account for a considerable proportion of the genome’s repetitive fraction. The satellite DNA families’ repeat profiling revealed a point mutation along the reference sequences. Limited TEs differentiation exists among geographically distant populations of *Spodoptera frugiperda*.

## 1. Introduction

The *Spodoptera frugiperda* (J.E. Smith) larvae (Lepidoptera: Noctuidae) is a polyphagous agricultural pest that attacks food and fiber staples like corn, cotton, sorghum, rice, and vegetable crops [1,2]. The fall armyworm has numerous hosts, the most common of which are 186 plant species from 42 families [3]. RNA sequencing and de novo transcriptome assembly were utilized to generate transcriptomes and conduct various analyses of *Spodoptera frugiperda* [4,5]. Similarly, multiple genomic DNA libraries were sequenced and used for genome analysis to perform genome assembly for *Spodoptera frugiperda*. For example, the ovary cells Sf21 were used for genomic DNA isolation and library preparation, and an assembly size of 358 Mb was reported for the *S. frugiperda* genome, which was 90% of the predicted size of 400 Mb [6]. Similarly, genome assembly was performed using whole-genome sequencing data from two male larvae of the corn strain and one larva of the rice strain, and two entirely distinct genome assemblies were reported for the corn strain (438 Mb) and the rice strain (371 Mb) [7]. Another study from China reported a chromosomal-level assembled genome of *S. frugiperda* with a size of 486 Mb using muscle tissue for DNA extraction and library preparation [8]. The disparity in genome size between species could result from numerous evolutionary processes [9]. Previous studies of repeated DNA sequence analysis reported different compositions of repetitive contents in *S. frugiperda* species, such as 29.16% in corn strains and 29.10% in rice strains [7], and accounting for 28.24% of the male genome in another study of chromosomal-level genome assembly from Yunnan, China [10]. Similarly, a draft genome of fall armyworm from India reported 20.28% of repeat content [6], and 28% repeat content were described during the genetic adaptations study of *Spodoptera frugiperda* from Zhejiang, China [8]. The *S. frugiperda* maize strain from Lusaka, Zambia, comprises 27.18% of repeated sequences [11]. Repetitive DNA sequences, including satellite DNAs, are responsible for genome size expansion and evolution [12,13,14,15]. Transposable elements account for between 50% and 90% of the DNA in higher plants’ genomes [16]. Retrotransposons, transposons, and satellite repeats are significant drivers in genome size fluctuation. Additionally, although mobile DNA is found in all living organisms, its abundance in the genome differs dramatically between yeast and pufferfish (by a few percent) and maize and barley (60–70 percent) [17]. Furthermore, there is a significant direct correlation between the size of a genome and the fraction of mobile DNA contained within it. Therefore, the pufferfish genome is minimal; meanwhile, the human genome and other known mammalian genomes are quite large, containing around 50% mobile DNA. Likewise, the type of mobile DNA that prevails in an organism’s genome varies significantly between species. In *Caenorhabditis elegans*, it ranges from over 90% DNA transposons to 100% of one type of retrotransposon in *Saccharomyces cerevisiae*, a budding yeast. Similarly, the human genome repetitive part comprises 75% of one type of retrotransposon [18]. It is unknown why one type of mobile DNA is accepted into the genome of one organism, but not another. Rapid and concerted evolution results in the formation of the genome- or species-specific sequences [19]. In the past, the transposable elements were considered as non-functional or junk DNA sequences, but are now believed to be the primary components of genome evolution [17]. Genetic reorganization processes such as inversion, translocation, fusion, fission, and the complete amplification or eradication of repetitive DNA sequences and gene-causing mutations have all contributed to genome evolution [20,21].

The analysis of the *Drosophila melanogaster* genome facilitated the first comprehensive classifications of the TE abundance, variety, age, and genome dispersion in an insect [22,23,24]. These investigations have elucidated how the completeness and quality of assembled genomes and the annotation methods have a significant effect on the estimation of TE diversity and abundance. When heterochromatic regions were added, and de novo TE annotation procedures were implemented, the TE content of the *D. melanogaster* genome increased from 2% in early studies of euchromatic domains to 15% [24,25]. The genetic distance computation revealed that most of the TE copies originated from the recent transposition events [26,27]. The TE content of insects varies significantly between mosquitoes (*Aedes aegypti*), Coleoptera (*Tribolium castaneum*), and Lepidoptera (*Bombyx mori*). In some species, DNA transposons and non-LTR retrotransposons predominate, varying from 6% in *T. castaneum* to 48% in *Aedes aegypti*. With the drop in sequencing costs, the number of genomics research projects that feature specific sections on TE annotation has increased [28,29,30]. TE content comparisons with an emphasis on surveying the evolution of TE content across genomes at scales ranging from a single insect genus to the entire arthropod phylum are now common [20,31,32,33,34,35,36]. Peccoud et al. automatically generated TE sequence annotation in 195 publicly available insect genomes [37]. Satellite DNAs have been identified in a few insect species using conventional methods (ladder pattern electrophoresis and genomic restriction digestion). In addition to the satellite DNA families identified using conventional methods, several additional satellite DNAs were identified in many species using a genomic approach [35,38]. Initially, the repeatome and satellitome were improperly described; however, next-generation sequencing (NGS) technologies have revolutionized the field of TEs analysis [13,39]. The power of next-generation sequencing (NGS) advanced technology to produce gigabytes of data in a solitary run empowers the investigation of highly complicated elements of repetitive DNA sequences in plants [40,41,42,43]. NGS platforms, such as Illumina sequencing, hasten the genome assembly process [44]. A large number of insects taxa lack the complete composition description of TEs and satellitome. The satellite DNAs and multi-gene families 5S and 45S rDNA, as well as the H3-H4 histone genes, have been reported in many species, and some of them are listed here, including grasshoppers *Locusta migratoria* (migratory locust) [14,45], *Eyprepocnemis plorans* (lamenting grasshopper) [46], *Abracris flavolineata* [47], *Dichroplus pratensis* [48], *Rhammatocerus brasiliensis* [49,50], *genus Isophya* [51], and in Coleoptera order *Tribolium castaneum* (Red flour beetle) [52]. Similarly, other studies have described the satellitome in detail for *Rhodnius prolixus*, *Triatoma infestans*, *Hippodamia variegate*, *Pyrgomorpha conica*, and *Holhymenia histrio* [53,54,55,56,57]. In this work, we used eight raw genome samples of *S. frugiperda* from eight different geographical locations for understanding the geographic impact on repetitive DNA sequences and diversity of dynamic satellite DNA of *S. frugiperda* genome. We compared satellite DNA diversity between samples and performed a genome-wide comparison of transposable elements between geographically distant *S. frugiperda* populations.

## 2. Materials and Methods

### 2.1. Raw Genome Samples Downloaded from NCBI

The raw genome data of the *S. frugiperda* species were obtained from the publicly accessible database NCBI. *S. frugiperda* genomic data from eight different geographical areas were chosen for satellitome and TEs investigation. The galaxy platform’s SRA server option was used to download all data (following an online tutorial) [58]. The raw genome samples were downloaded from NCBI with following SRR numbers: ZHJ = SRR11528381, BRA = SRR12044617, USA = SRR12044620, PUE = SRR12044635, ARG = SRR12044656, CAS = SRR12072096, FRA = SRR12701296, and KEN = SRR12044648.

### 2.2. Pre-Processing of the Sequenced Data, Quality Control, and Random Sampling

The recommended genome coverage of 0.01–0.5× and pair-end reads data were used for RepeatExplorer2 analysis. We have used the SeqTK tool v1.3 (https://github.com/lh3/seqtk, accessed on 1 September 2021) to perform random sampling and extracted 5 million reads from each sample for repetitive analysis, guaranteeing that each selected sample encompasses the entire genome. The raw genome data were uploaded to the Repeatexplorer2 galaxy server. The data quality was tested using the FastQC tool, integrated inside the RepeatExplorer2 Galaxy instance. Fastq files were pre-processed using the RepeatExplorer Galaxy platform’s “pre-processing of fastq paired reads” tool with the default settings. Trimming, read quality filtering, removing single reads while maintaining entire pairs, cut-adapt filtering, and fastq interlacing are all part of the pre-processing step.

### 2.3. The Clustering Analysis Using RepeatExplorer2 and TAREAN Tool

The comparative analysis was conducted in accordance with the protocol highlighted by Novak et al. [59]. RepeatExplorer2 utility tool “FASTA read name affixer” was used to assign three-letter, species-specific prefixes to the read names. The first three capitalized letters of each geographical location name were used as a prefix, and the remaining settings were left unchanged. Then, in RepeatExplorer Utilities, we used the “Read Samples” function to perform additional sub-sampling. We chose the interlaced Fasta files and set each file’s “number of reads” to 500,000, as well as the random seed number to 10. Using the “Text manipulation—Concatenate datasets” tool, all three species datasets were concatenated. After selecting and inserting all three species data with the formatted reads in the correct order, we ran the tool. The FASTA file was concatenated with 4 million reads, 500,000 reads from each sample.

Finally, the clustering analysis was carried out in RepeatExplorer2 and the TAREAN tool using the concatenated FASTA file retrieved in the previous step as an input file. The comparative mode of RepeatExplorer2 clustering was set to the following parameters: pair-end reads = yes, sample_size = 4 million reads, reference database = metazoa 3, and select queue = “long”. The options comparative analysis = “yes”, custom database = “Repbase”, and group code length = “3” were set in the advance settings. For the tandem repeat analyzer (TAREAN), we used the default parameters of sample size = 1.5 million reads and queue = basic and fast. An HTML archive report, a log file, and an HTML report were all generated as the clustering analysis output. HTML archive reports were downloaded for a more thorough examination. The two output files from the RepeatExplorer pipeline (https://github.com/kavonrtep/revis, accessed on 1 October 2021) were used to run the script “plot comparative clustering summary.R” to generate a comparative visualization of transposable elements (TEs) results.

### 2.4. Homology Searches against Publicly Available Databases

The unclassified clusters from the Repeatexplorer output with spherical or circle graphs were uploaded to the YASS software web server (https://bioinfo.lifl.fr/yass/index.php, accessed on 1 October 2021) for tandem repeat detection [4]. Similarly, we tried to distinguish and classify satellite DNA families into subfamilies based on the similarities using the ‘rm homology.py’ script from the satminer toolkit (https://github.com/fjruizruano/satminer, accessed on 1 November 2021). Each individual of the satellite DNA family was given a name based on the nomenclature established by Ruiz-Ruano et al. [14]. The first letter of the genus and two letters from the species names were used to assign a specific satellite DNA family. The word “Sat” from the satellite was added to the name, followed by the length of a particular monomer, e.g., (SfrSat01-126). The homology searches for each satellite DNA family were performed using the Censor tool (http://www.girinst.org/, accessed on 1 December 2021) against the transposable elements databases. We used filtering by selecting the arthropod part, rather than the entire database, to be more precise. Then, we searched all databases for any similarities to SatDNA consensus sequences. The BLAST tool was also used to discover each satellite DNA family for similarities or coding sequences against the D-fam and NCBI databases.

### 2.5. Comparative Satellitome Analysis

RepeatMasker v4.1.1 (http://repeatmasker.org, accessed on 1 December 2021) was used to determine the satellite DNA families’ abundance and divergence from the monomer consensus sequence with the following parameters: “-a” and search engine RMblast. We randomly chose 2 million reads and used the customized library (-lib) flag to map them against the entire collection of satellite DNA consensus sequences. We used the “calcDivergenceFromAlign.pl” script to determine the average deviation for each sample and the “createRepeatLandscape.pl” script from the RepeatMasker package to produce a satellite DNA landscape.

### 2.6. The Usage of the DANTE Tool for Extracting Consensus Sequences of TEs

The DANTE tool implemented on the RepeatExplorer Galaxy Platform was used to extract the consensus sequences of transposable elements. The contigs were extracted from the RepeatExplorer2 result archive file using the script ‘Extract contig from RepeatExplorer2 archive’, which is available under the ‘RepeatExplorer Utilities’ option. This FASTA-formatted contig file was used as input for the DANTE tool, selecting the metazoan database, the scoring matrix ‘BLOSUM80’, and zero iterative searches. This step produced three files: filtered output for protein domains (Fasta format), filtered output for protein domains (gff3 format), and total output (gff3 format). The subsequent step used the ‘Extract Domains Nucleotide Sequences’ tool to obtain the final consensus sequences for each TE. This final consensus TEs file was manually renamed and utilized as the FASTA input file for the Repeat Profiler program.

### 2.7. Satellite DNA Families Repeat Profiling Analysis

The repeat profiling analysis for each satellite DNA, TEs, and rDNA were done using RepeatProfiler software release 1.1 (https://github.com/johnssproul/RepeatProfiler, accessed on 1 January 2022). The short-read, low coverage, raw genome data is used to generate, compare, and visualize repeat elements profiles. We used FASTA files from satellite DNA and TEs as a reference sequence for this repeat profile analysis, mapping against 5 million randomly chosen reads from each sample. The correlation analysis flag was activated for comparing each satellite DNA profile against eight geographical locations samples. The user-groups.txt file containing the samples information was necessary for the correlation analysis. We utilized the “pre-corr” flag to obtain this file and manually assigned the group number to each sample [60].

## 3. Results

### 3.1. The Spodoptera Frugiperda Species Repeat Composition and Its Comparative Visualization

**Figure 1:**
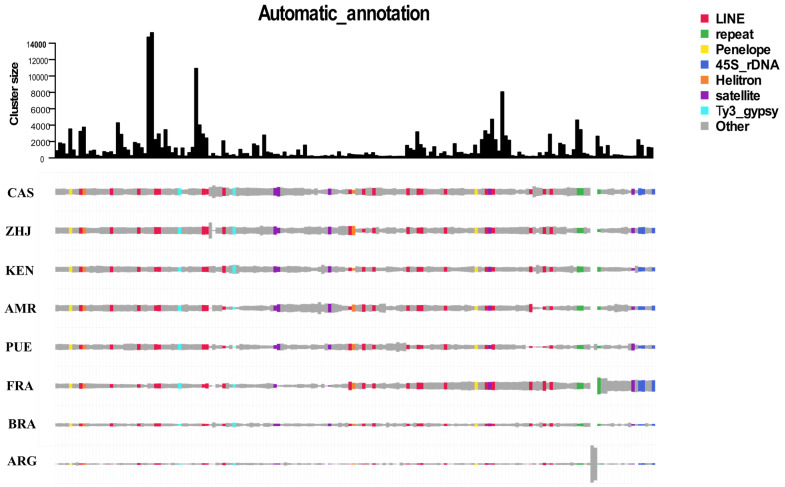
A comparative repeat graph of geographically distant populations of Spodoptera frugiperda. The total number of reads in individual clusters are depicted in the top bar plot. The size of the rectangle is equal to the number of reads in a cluster for each sample. The clusters and samples were sorted using hierarchical clustering. The final annotation of the clusters from Repeatexplorer2 results were used to color unique rectangles.

The top bar graph represents a particular family of repeats, and the graph’s height denotes the depth of reads clustered in it. The color rectangles reflect the total number of reads from different samples. Most of the top clusters have similar-colored rectangle heights, indicating that TEs are commonly shared across all samples. The commonly shared TEs include LTR, LINE, penelope, and other repeat elements families. The two unique, unclassified repeat clusters were observed in the ARG species compared to all remaining samples (see Figure 1).

**Figure 2:**
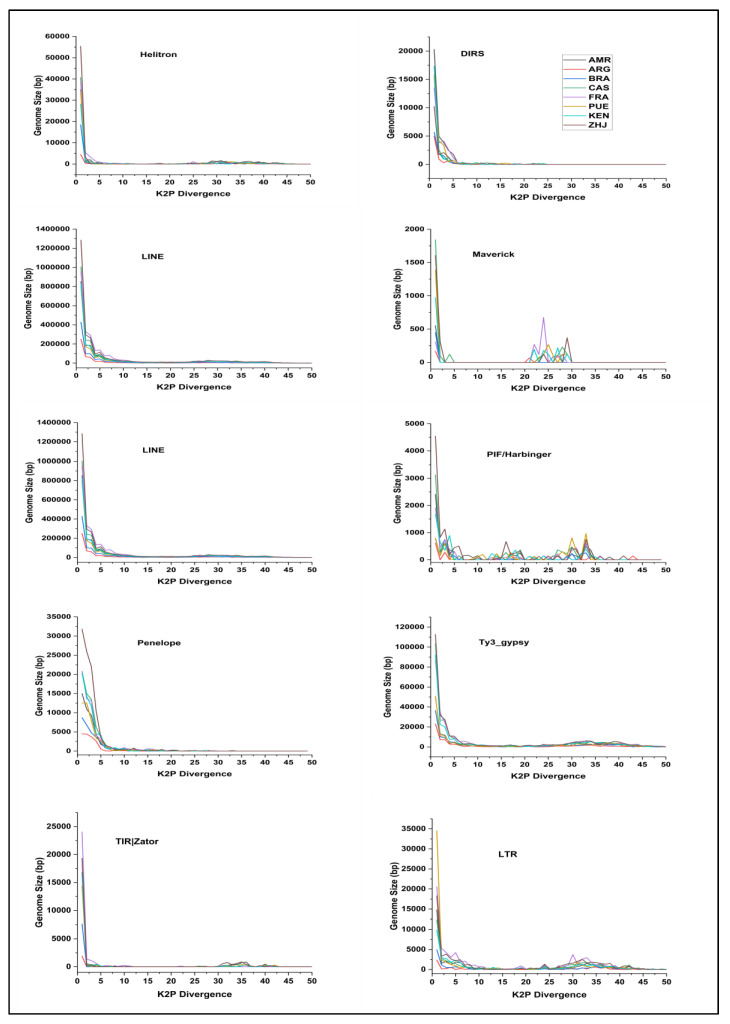
The graph represents the overall repeat landscape of Spodoptera frugiperda’s various TEs families from eight different geographical locations. The consensus sequences of each family were extracted using the DANTE tool.

The repeat landscape demonstrates unambiguously that the LINE and Penelope elements dominate the *Spodoptera frugiperda* species. Although most TEs sequences were only slightly divergent from the consensus sequences, a few were highly divergent, indicating the presence of older and more variable sequences. We computed the average deviation of transposable elements mapping against the specified read samples using Repeatmasker. The Maverick and PIF/Harbinger elements displayed the most sequence divergence. Both elements showed a double peak pattern on the repeat landscape, indicating the presence of both recently active and older repeat residues within the genome. The newer/recent copies elements peak was observed inside the 5% and another peak for older copies was observed at 30–35% divergence. However, the contribution of older, highly divergent repeats to genome size was much lower than recent active repeats. The recent copies of all elements contributed to genome size at a higher rate in Spodoptera frugiperda species. Helitrons, LTR Ty1 copia, LTR Ty3 gypsy, LINE, bel-pao, Penelope, and TIR Sola had the lowest amount of sequence divergence, with divergence rates ranging from 0% to 5%. The sequences with the lowest amount of deviation have made a significant contribution to the genome size of their respective samples. Overall, the transposable elements families found in common across all eight geographical samples had a similar amount of divergence, distribution, and genome size proportion (see Figure 2).

### 3.2. Age Calculation of Transposable Elements

We conducted an age analysis on each TEs element to determine how many million years old sequences are present in the *Spodoptera frugiperda* species. The TE landscapes are represented as ‘1 My bins’ along the *x*-axis. The genome size portion is represented along the *y*-axis, both of which are inferred from the RepeatMasker align output. It was discovered that most families have younger elements than older members. The young elements are between 0 and 15 million years old, and they contribute more to genome size expansion than the older elements. The LINE, Ty3_gypsy, Ty1_copia, Penelope, and DIRS families primarily comprise young elements compared to the older elements found in the *Spodoptera frugiperda* genome. The Maverick elements showed a double peak pattern on repeat landscapes; nevertheless, when the evolutionary age of this family was estimated, it was discovered that the majority of elements belonged to younger elements rather than older elements (Figure S1).

The majority of the sequences ranged from 1–5 million years old. Similarly, LTR Ty1-copia ranged in age from 0–10 million years; Penelope, with a few older sequences, ranged from 1–15 million years; and Maverick’s age ranged from 0–10 million years. On the other hand, while most Bel-pao sequences were young in evolution (ranging from 0 to 10 million years old), across all samples, a few rDNA elements were as old as 35–40 million years.

### 3.3. The Generalized Structure of Transposable Elements

**Figure 3:**
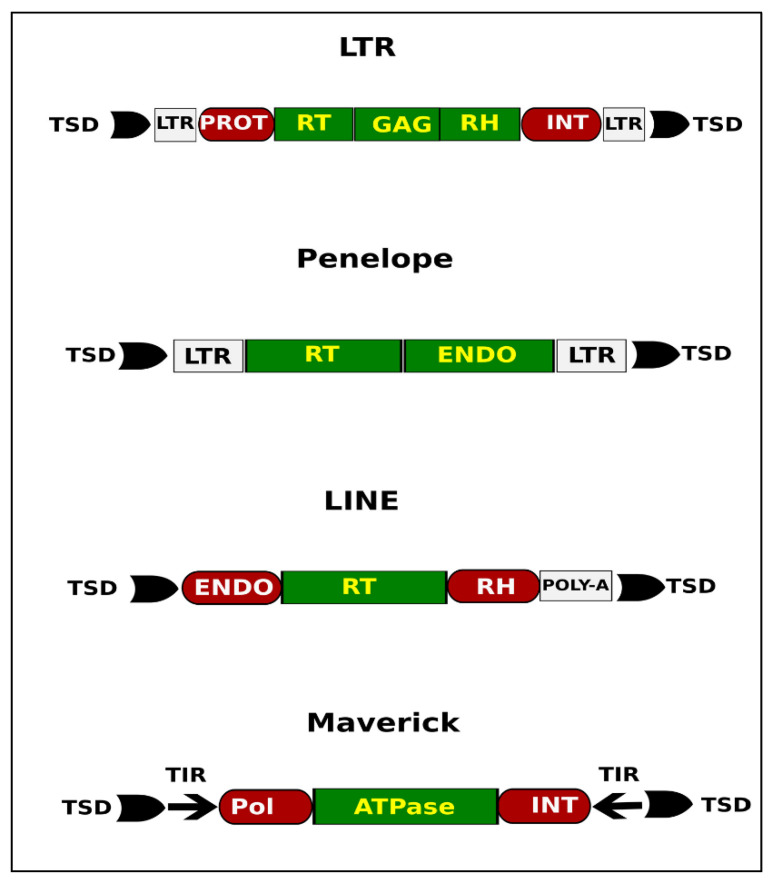
A generalized structure of TEs families present in the *Spodoptera frugiperda* genome. Protein domains for each family were extracted using the DANTE tool.

Long terminal repeat (LTR) retrotransposons have LTRs that range in length from several hundred to several thousand base pairs. In addition to the gag gene, LTR retrotransposons contain the pol gene, which codes for reverse transcriptase, ribonuclease H, and integrase. Reverse transcription occurs inside a viral-like particle’s cytoplasm (GAG). The presence of two direct repeats flanking the element’s core region (5′ LTR and 3′ LTR) is a common feature of LTR-retrotransposons. Each LTR-retrotransposon family generates a TSD with a unique length. We discovered that LTR Ty3-gypsy elements contain RT (reverse transcriptase), RH (ribonuclease H), INT (integrase), and PROT (protease) protein domains (Figure S2). Penelope elements present in the *Spodoptera frugiperda* genome contain two protein domains, RT (reverse transcriptase) and ENDO (endonuclease), and two direct repeats flanking the core region of the element and target site duplication (TSD). The LINE elements include three protein domains, RT (reverse transcriptase), RH (ribonuclease H), and ENDO (endonuclease), a poly-A tail flanked and target site duplication (TSD) (Figure S3). Mavericks are powerful transposons that have been discovered in different eukaryotic lineages. They encode various numbers of proteins that include DNA polymerase and an integrase. In our results, Maverick contains three protein domains, ATPase, INT (integrase), and POL (a gene that encodes for reverse transcriptase), along with the common feature of TIR (terminal inverted repeat) flanked by TSD (target site duplications) (Figure 3).

### 3.4. The Classification of Satellite DNA Families and Sequence Similarity Searches

A total of seven satellite DNA families were discovered using the TAREAN software implemented on the Galaxy platform. The dynamic nature of satellite DNA induces monomer variations, and we searched for satellite DNA homology to each other and classified them into completely separate superfamilies. However, we discovered no satellite DNA family with over 50% similarity. The homology searches revealed that the satellite DNA family SfrSat05 contains the insertion of Helitron elements (Helitron-1_Etal and Helitron-4a_Diul) that traced back to the genus Heliconius species. A long fragment of Helitron-1_Etal elements (302 bp) was detected in the SfrSat05 family, aligning along the consensus sequence position 90 to 402 bp. Similarly, the 229 bp long fragment of Helitron-4a_Diul aligned to the consensus sequence position 285 to 526 bp (Figure S4). Helitron insertion could have aided in the genome’s homogenization and fixation of SfrSat05.

### 3.5. Comparison of Satellitome Landscapes

**Figure 4:**
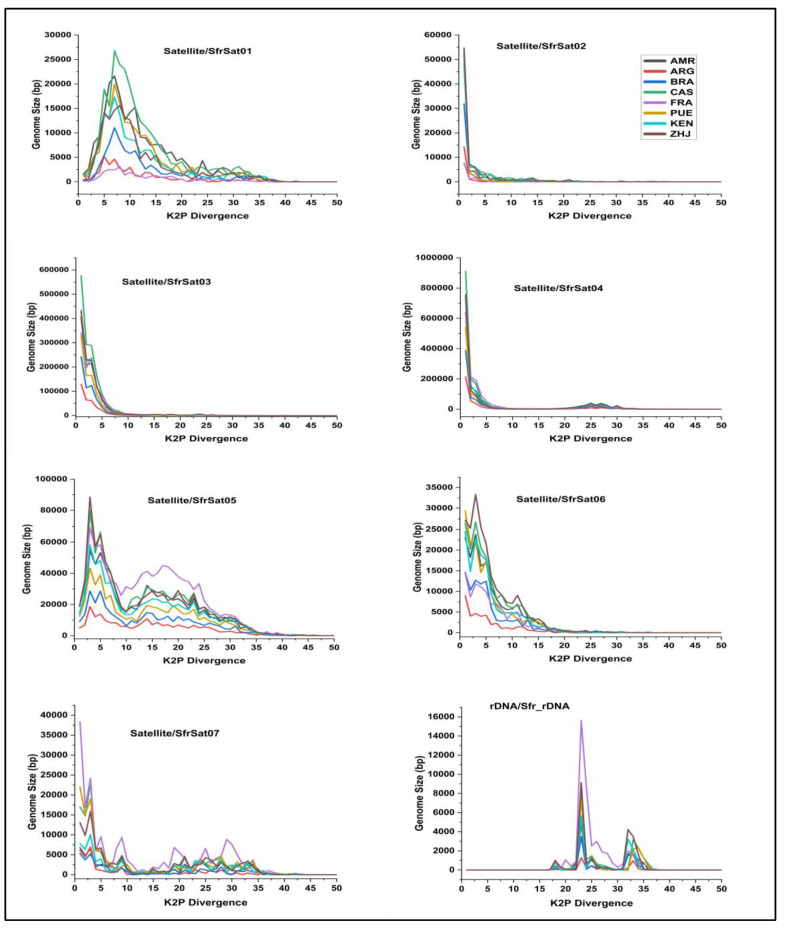
The graph denotes the overall repeat landscape of Spodoptera frugiperda different satellite DNA families from eight different geographical locations. The consensus sequences of each family were obtained from the RepeatExplorer2 results archive file.

The most recent or active copies of repeat elements are clustered on the left side of the repeat landscape, while older or degraded copies are clustered on the right. The satellite DNA families with the highest peaks of genome proportion below 5% genetic divergence did not deviate from the reference and represent the process of genome homogenization. Five satellite DNA families showed a two-peak trend, which reflects monomer variations and the presence of two subunits of a specific repeat. The SfrSat01, SfrSat05, SfrSat06, and SfrSat07 repeat sequences disclosed two peaks in *Spodoptera frugiperda* genome, indicating minimal divergence and around 15% divergence (Figure 4).

### 3.6. Estimating the Abundance, Divergence, and Copy Number of Satellite DNA Families

The satellitome analysis of the *S. frugiperda* genome revealed that the monomer size of satellite DNA families varies 108 nt for SfrSat06-108 to 1824 nt for the SfrSat07-1824 family. Similarly, the satellite DNA family’s A plus T content varies from 49 to 73%, with a median of 61%. Only one G plus C rich satellite DNA family, SfrSat02, was discovered, with a value of 51%. (Table 1). We observed no substantial relation between monomer length and A plus T content (Spearsman correlation test: rs = 0.18, t = 0.40, *p* = 0.70) supporting information (Table S1).

The abundances of satellite DNA families in *S. frugiperda* ranged a minimum of 0.01 for SfrSat07 to a maximum of 0.26% for the SfrSat04 family. Similarly, the total share of seven satellite DNA families accumulated in the genomes was 0.65%. The satellite DNA families SfrSat04 and SfrSat03 account for more than half of the overall satellite DNA abundance in the *S. frugiperda* genome. The SfrSat03 and SfrSat04 satellite DNA families were abundant among all seven satellite families. There was no conspicuous association observed between monomer repeat unit size and abundance (rs = 0.14, t = 0.22, *p* = 0.72).

On average, the *S. frugiperda* satellite DNA families’ kimura genetic divergence ranged from a minimum of 1.89% for SfrSat02 and a maximum of 13.2% for SfrSat05. The other satellite DNA families have shown a moderate K2P genetic divergence of 2.45%, 5.25%, 5.05%, 10.74%, and 12.06% in SfrSat03, SfrSat04, SfrSat06, SfrSat01, and SfrSat07, respectively (see Table 1). The K2P divergence and monomer length or satellite family abundance did not reveal any positive correlation ((rs = 0.50, t = 1.29, *p* = 0.25) and (rs = 0.17, t = 0.40, *p* = 0.70), respectively) (Table S1).

### 3.7. Satellite DNA Families Repeat Profiling Present in Spodoptera Frugiperda

**Figure 5:**
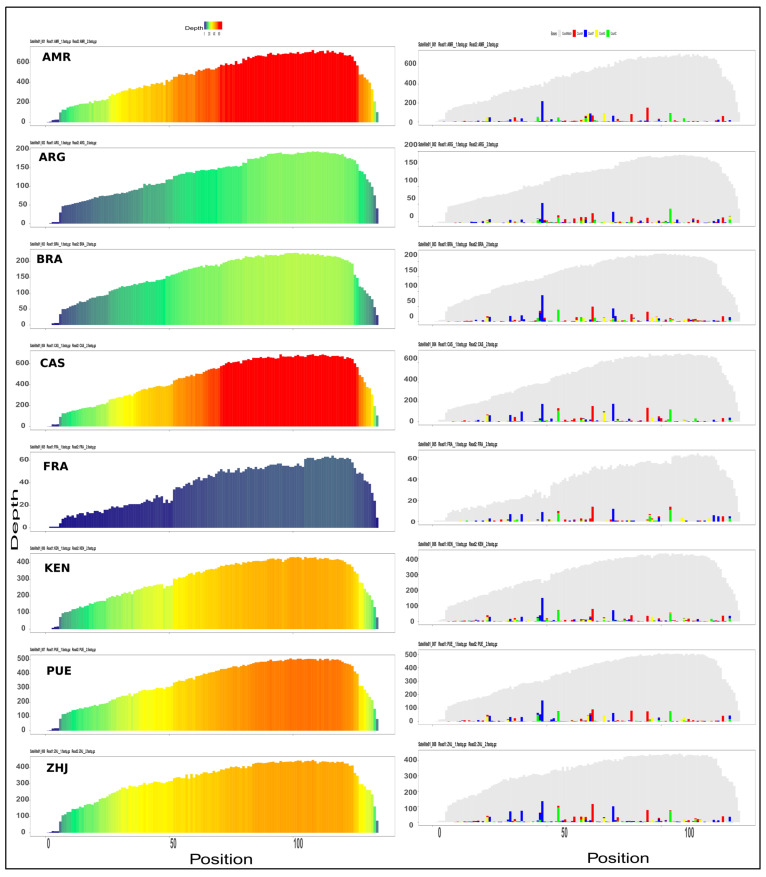
The colour-enhanced and variant profiles of the SfrSat01 satellite DNA family against eight different geographical location samples.

**Figure 6:**
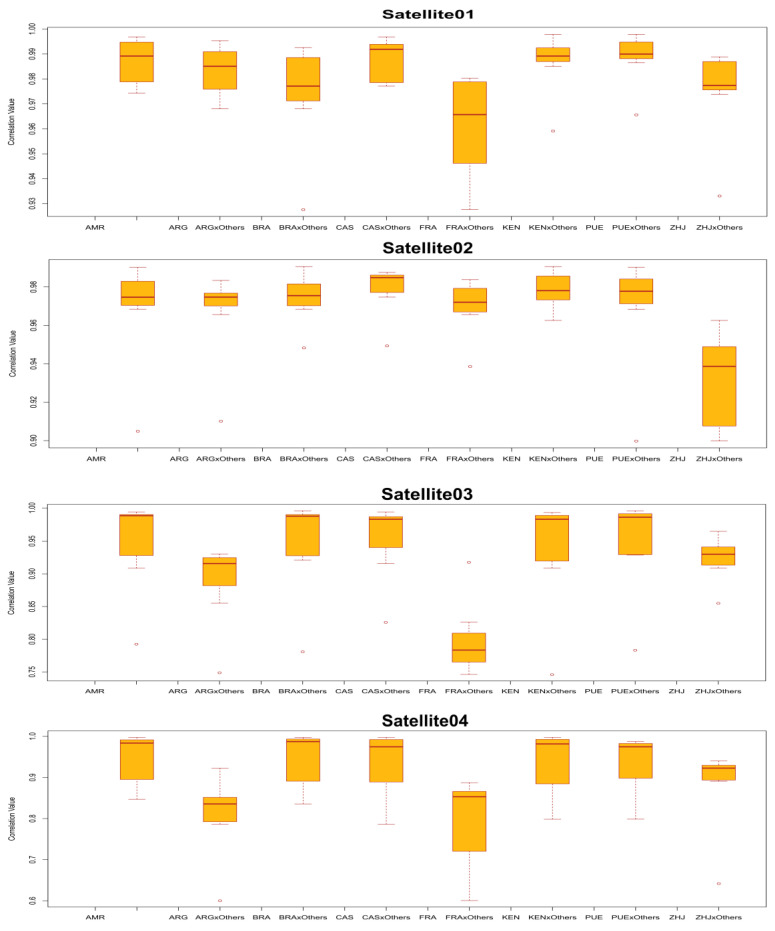
The correlation analysis of individual (top four) satellite DNA family comparisons across the three samples of each species, showing similar correlation values within samples and varying in between group correlation values.

The color enhanced profile of the SfrSat01 satellite DNA family revealed similar read depth coverage across all the samples, but lower coverage for geographical location FRA. The high and low read depth coverage of profiles depends on the deviation of sequences from the reference, which is reflected in the variant profiles of the family (Figure 5). The species-specific signature of the SfrSat03 satellite DNA family is more evident across all the samples of the *Spodoptera frugiperda* genome, where the consensus sequence repeat profile has shown point mutation at two sites of monomer positions (284 and 285) and 322 (see Figure 5 and Figure S6). However, we inferred that these base pairs were deleted due to deletion mutation. Like variant profile graphs, the enhanced color profile of SfrSat02, SfrSat04, SfrSat05, SfrSat06, and SfrSat07 was identical in all the sample s (Figures S5 and S7). Similarly, the small differences in satellite DNA family profiles observed across all samples is associated with changes in repeat abundance and sequence divergence compared to the consensus sequence Figures S8–S10). Correlation analysis produces boxplots that illustrate a high degree of correlation between groups. Correlation analysis of each group for all references provided a detailed depiction of how all reference sequences are correlated both within and between groups. The correlation coefficients between groups for the SfrSat01 family ranged from 0.97 to 0.99 for FRA and CAS samples, respectively. Similarly, the SfrSat03 and SfrSat05 satellite DNA families have displayed moderate correlation values for the sample FRA, but SfrSat07 has shown the lowest value (0.50) for ARG as compared to all other samples (Figure S11). The primary reason for these higher correlation values is that all samples belonged to the same species but were geographically distant populations (Figure 6).

## 4. Discussion

### 4.1. Repeat Sequences Composition and Diversity among the Genomes

The transposable elements with similar abundance and divergence were shared across the samples, displayed in the top clusters. The prominent shared TEs comprise LINE, Penelope, maverick, LTR, and other unclassified repeat elements. In our study, the repetitive DNA constitute 21% of the whole genome of *S. frugiperda*. Previous studies of repeated DNA sequence analysis reported different compositions of repetitive contents in the *S. frugiperda* species, such as 29.16% in corn strains and 29.10% in rice strains [7] and accounting for 28.24% of the male genome in another study of chromosomal-level genome assembly from Yunnan, China [10]. Similarly, a draft genome of fall armyworm reported 20.28% of repeat content [6], and 28% repeat content were described in a recent genome assembly [8]. Repetitive DNA sequence, including satellite DNAs, are responsible for genome size expansion and evolution [12,13,14,15]. When compared to the hymenopterans studied, the genomes of termites and cockroaches have a larger level of repetitive DNA. The repetitive elements constitute almost 55% of the total genome in *B. germanica* and *Cryptotermes secundus* [61], double the 28% *Zootermopsis nevadensis* genome, and greater than 46% of the higher termite *Macrotermes natalensis* [62,63]. The *B. germanica Zootermopsis nevadensis*, *Macrotermes natalensis*, and *C. secundus* genomes dominated by LINEs elements, especially the BovB subfamily, indicated the amplification of LINEs elements in common ancestors, such as Blattodeas. Likewise, the amplification of LINE and LTR elements were observed in the genome of *Spodoptera frugiperda*. The transposable elements comprise 16% and 14.6% of the total *Helicoverpa zea* and *Helicoverpa armigera* genomes [64]. Similarly, 35% and 25% repetitive sequences were discovered in the *Bombyx mori* (silkworm) and *Heliconius melpomene* (postman butterfly) genomes [65]. The genome of *Drosophila melanogaster* comprises 20% of all TEs [66,67]. The repetitive DNA sequences varied significantly across the insects’ orders depending on the genome sizes. The *Apis mellifera* (honey bee) genome contains 8–10% repetitive elements [68]. In contrast, the *Tribolium castaneum* genome consists of over 42% of transposable elements [30,69]. In the blister beetle *Mylabris aulica*, the repetitive sequence made up 50.62% of the overall length. In comparison to *Mylabris aulica* genomes, the *H. cichorii* and *H. phaleratus* species contain 22.73% and 13.47% repetitive content, respectively [70]. The mammals transposable elements acquisition evolutionary pathways are conserved across species regardless of clade-specific discrepancies in TE composition, probably due to certain shared traits [71]. Satellite DNA repeats, the fast-evolving component of repetitive DNA in evolution, have varied fractionally between samples. Our findings corroborated those of [72,73], indicating that insects’ genomes, particularly the Lepidoptera, are dominated by LTR and LINE elements.

### 4.2. Satellitome Analysis and Satellite DNA Family Number Differences

The increase in the genome size of the *Calliptamus barbarus* species was linked to the proliferation of species-specific satellite DNA families, highlighting the importance of satellite DNA in genome evolution [74]. According to the contradictory findings, satellite DNA families were not responsible for genome size evolution [75]. These studies made the dynamic satellite DNA analysis crucial for investigating genome size evolution across closely related species. A diverse set of satellite DNA families presents in different insect orders genomes such as 9 in the *Tribolium castaneum* genome [76], up to 16 in *D. melanogaster* [77], 76 in the grasshopper species *P. conica* [54], and 62 in the migratory locust *L. migratoria* [14]. Similarly, the *Eneoptera surinamensis* genome consists of 45 satellite DNA families [78], and there are 29 in *Hippodamia variegata* (Ladybird Beetle) [53], 53 in grasshopper *Ronderosia bergii* [72], and 56 to 92 satellite DNA families across the four species of morabine grasshoppers [12]. Likewise, *Rhammatocerus brasiliensis*, *Schistocerca rubiginosa*, and *X. d. angulatus* possess 12, 9, and 18 satellite DNA families [79]. In the three species studied, a total of seven putative satellite DNAs were discovered: one in *Cydalima perspectalis*, two in *Diatraea postlineella*, and four in *Ostrinia nubilalis* [80]. Likewise, 42 satellite DNA families were reported in the repeatome analysis of *Triatoma infestans* [55]. Twenty-nine (29) and 20 satellite DNA families have been identified in *Tagasta tonkinensis* and the genus *Calliptamus* species, respectively [74]. Similarly, we discovered seven (7) satellite DNA families in the genome of *Spodoptera frugiperda*, the majority of which were shared across all samples.

### 4.3. Satellite DNA Families, Repeat Profiling, Double-Peak Pattern, and Monomer Size Variation

The satellite DNA families’ monomer-length varies between 108 bp SfrSat06-108 to the second 1824 bp largest SfrSat07-1824 satellite family recorded in Lepidoptera. The satellite DNA family’s A plus T concentrations ranged from 49 to 73%, with a median value of 61%. Only one satellite DNA family, SfrSat02, was an observed G plus C-rich family with a value of 51%. Similar work is reported, describing monomers of the satellite DNA that were highly variable in size, ranging from 123 bp to the largest satellite DNA family in Lepidoptera with 2244 bp [80]. Satellite DNA families with varying monomer lengths have been reported in insect order orthoptera genomes, including the 320 bp PcoSat25A-320 satellite DNA family in *P. conica* [54], the 784 bp RbeSat14-784 family in *R. bergii* [72], and the 400 bp *L. migratoria* largest family LmiSat05–400 [14]. In addition, satellite DNA families with the largest monomer sizes have been discovered in a wide range of insect species, including a 2,5 kb monomer size satellite DNA family in the ant *M. subopacum* [81], a PStl family of 1169 bp in *M. goudati* [82], and a HvarSat07-2000 family of 2000 bp in *H. variegate* [53]. The variation in monomer size of satellite DNA families does not affect copy number and A plus T richness. There was no direct association between kimura divergence (genetic divergence) and the monomer size of satellite families in the genus *Calliptamus* species [74]. Our results were consistent in that genetic divergence has not shown a direct association against monomer size and abundance of each satellite family ((rs = 0.50, t = 1.29, *p* = 0.25) and (rs = 0.17, t = 0.40, *p* = 0.70), respectively). The SfrSat01, SfrSat05, SfrSat06, and SfrSat07 repeat sequences disclosed two peaks in the *Spodoptera frugiperda* genome, one indicating minimal divergence and the second around 15% divergence. 

This trend of double peaks is not unexpected, as the VspSat01-59 family of the fern *V. speciosa* and HvarSat01-277 family of *H. variegate* both demonstrated two distinct types of divergent repeats on a satellitome landscape. Likewise, the *C. italicus* and *C. barbarus* genomes’ satellite family CSat01-800 also revealed two peaks, one indicating very low divergence and the other denoting nearly 17% of divergence [53,74,83]. The species-specific signature of the SfrSat03 satellite DNA family is more evident across all the samples of the *Spodoptera frugiperda* genome, where the consensus sequence repeat profile has shown point mutation at two sites of monomer positions ((284 and 285) and 322). Similar results for the 5S-rDNA-02 family of genus *Calliptamus* and satellite DNA family CharSat01-52 in *Hemiodus gracilis* and *Brycon orbignyanus* have been reported previously [84]. Unequal crossing over and gene conversion across repeated DNA sequences can result in the concerted evolution of repeats within species and rapid stabilization of species differences [85]. Uneven coverage of repeats with sharp borders may be due to the differential amplification of truncated/fragmented repeat copies, reflecting the distribution of novel satellite DNA sequences or recent TE activity [86].

## 5. Conclusions

Our findings suggest that the satellite DNA families identified in *Spodoptera* *frugiperda* account for a considerable proportion of the genome’s repetitive fraction. Seven satellite DNA families were discovered, accounting for approximately 0.65% of the entire genome of *Spodoptera* *frugiperda* (fall armyworm). The TEs age analysis revealed that most TEs families consist of young copies that are 1–15 million years old; however, PIF/Harbinger has some older/degenerated copies of 30–35 million years old. The young/new elements contribute to genome size more than the older ones. We reported the protein domains present in a generalized structure of different TEs families. The satellite DNA families’ repeat profiling revealed the mutation process responsible for SfrSat03 satellite DNA variation. The limited transposable elements differentiation occurs among geographically distant populations of *Spodoptera* *frugiperda*. 

## Figures and Tables

**Table 1:** The divergence %, abundance %, and copy number for *Spodoptera frugiperda* estimated by Repeatmasker and the A plus T percentage of each satellite DNA family.

Satellite Name	Monomer Length	Avg. A + T %	Avg. K2P %	Avg. % Abundance	Avg. Copy_Number
SfrSat01	134	61	10.74	0.028	107,665.39
SfrSat02	156	49	1.89	0.0116	38,897.55
SfrSat03	524	73	2.45	0.201	201,474.44
SfrSat04	441	71	5.25	0.2313	276,294.85
SfrSat05	526	65	13.02	0.1313	132,916.06
SfrSat06	108	55	5.04	0.0263	128,961.93
SfrSat07	1824	51	12.06	0.0194	5827.51

## Data Availability

The raw genome data are already available and can be accessed from NCBI with following SRR numbers: ZHJ = SRR11528381, BRA = SRR12044617, USA = SRR12044620, PUE = SRR12044635, ARG = SRR12044656, CAS = SRR12072096, FRA = SRR12701296, and KEN = SRR12044648.

## Supplementary Materials

The following supporting information can be downloaded at: https://www.mdpi.com/article/10.3390/life12040521/s1, Figure S1: The interspersed repeat landscape of different transposable elements family in *Spodoptera frugiperda*; Figure S2: The genome browser output displaying the annotation tracks of different transposable elements protein domains sequences against the assembled genome of *Spodoptera frugiperda*; Figure S3: The genome browser output displaying the annotation tracks of LTR elements protein domains sequences against the assembled genome of *Spodoptera frugiperda*; Figure S4: The SfrSat05 family revealed sequence similarity to genus Heliconius species Helitron elements (Heltron-1_Etal and Helitron-4a_Diul); Figure S5: The colour enhanced and variant profiles of SfrSat02 satellite DNA family against eight different geographical location samples; Figure S6: The colour enhanced and variant profiles of SfrSat03 satellite DNA family against eight different geographical location samples; Figure S7:The colour enhanced and variant profiles of SfrSat04 satellite DNA family against eight different geographical location samples; Figure S8: The colour enhanced and variant profiles of SfrSat05 satellite DNA family against eight different geographical location samples; Figure S9: The colour enhanced and variant profiles of SfrSat06 satellite DNA family against eight different geographical location samples; Figure S10: The colour enhanced and variant profiles of SfrSat07 satellite DNA family against eight different geographical location samples; Figure S11: The correlation analysis of individual satellite DNA family (5 to 7) comparison across the three samples of each species shown similar correlation values within samples and varying in between group correlation values; Table S1: Table contains the spearsman rank order correlation test values to infer the possible correlation between the monomer length against the A + T content, K2P divergence, percentage abundance and copy number.
