# Supplementary material for: Satellitome Analysis and Transposable Elements Comparison in Geographically Distant Populations of Spodoptera frugiperda
Source: Life (Basel). 2022 Mar 31;12(4):521. doi: 10.3390/life12040521 (PMC9026859; doi:10.3390/life12040521)
Supplement: Supplementary file 1 [file life-12-00521-s001.zip › life-1638621-supplementary.pdf]

life

MDPI

Article

Satellitome Analysis and Transposable Elements Comparison in Geographically Distant Populations of *Spodoptera frugiperda*

Inzamam Ul Haq [1,†], Majid Muhammad [2,†], Huang Yuan [2], Shahbaz Ali [3], Asim Abbasi [4], Muhammad Asad [5], Hafiza Javaria Ashraf [6], Aroosa Khurshid [1], Kexin Zhang [1], Qiangyan Zhang [1] and Changzhong Liu [1,*]

1 College of Plant Protection, Gansu Agricultural University, Lanzhou, No. 1 Yingmen Village, Anning District, Lanzhou 730070, China; inzamam@st.gsau.edu.cn (I.U.H.); aroosakhurshid3@gmail.com (A.K.); 1120425163@qq.com (K.Z.); zhangqiangyan2@163.com (Q.Z.)
2 College of Life Sciences, Shaanxi Normal University, Xi'an 710100, China; majidento07@snnu.edu.cn (M.M.); yuanh@snnu.edu.cn (H.Y.)
3 Department of Agricultural Engineering, Khwaja Fareed University of Engineering and Information Technology, Rahim Yar Khan 64200, Pakistan; shahbaz@kfueit.edu.pk
4 Department of Zoology, University of Central Punjab, Bahawalpur Campus, Bahawalpur 63100, Pakistan; asimuaf95@gmail.com
5 College of Life Science, Fujian Agriculture and Forestry University, Fuzhou 350002, China; axadch@fafu.edu.cn
6 College of Plant Protection, Fujian Agriculture and Forestry University, Fuzhou 350002, China; hafizajavaria@yahoo.com
* Correspondence: liuchzh@gsau.edu.cn
† These authors contributed equally to this work.

Supplementary Materials

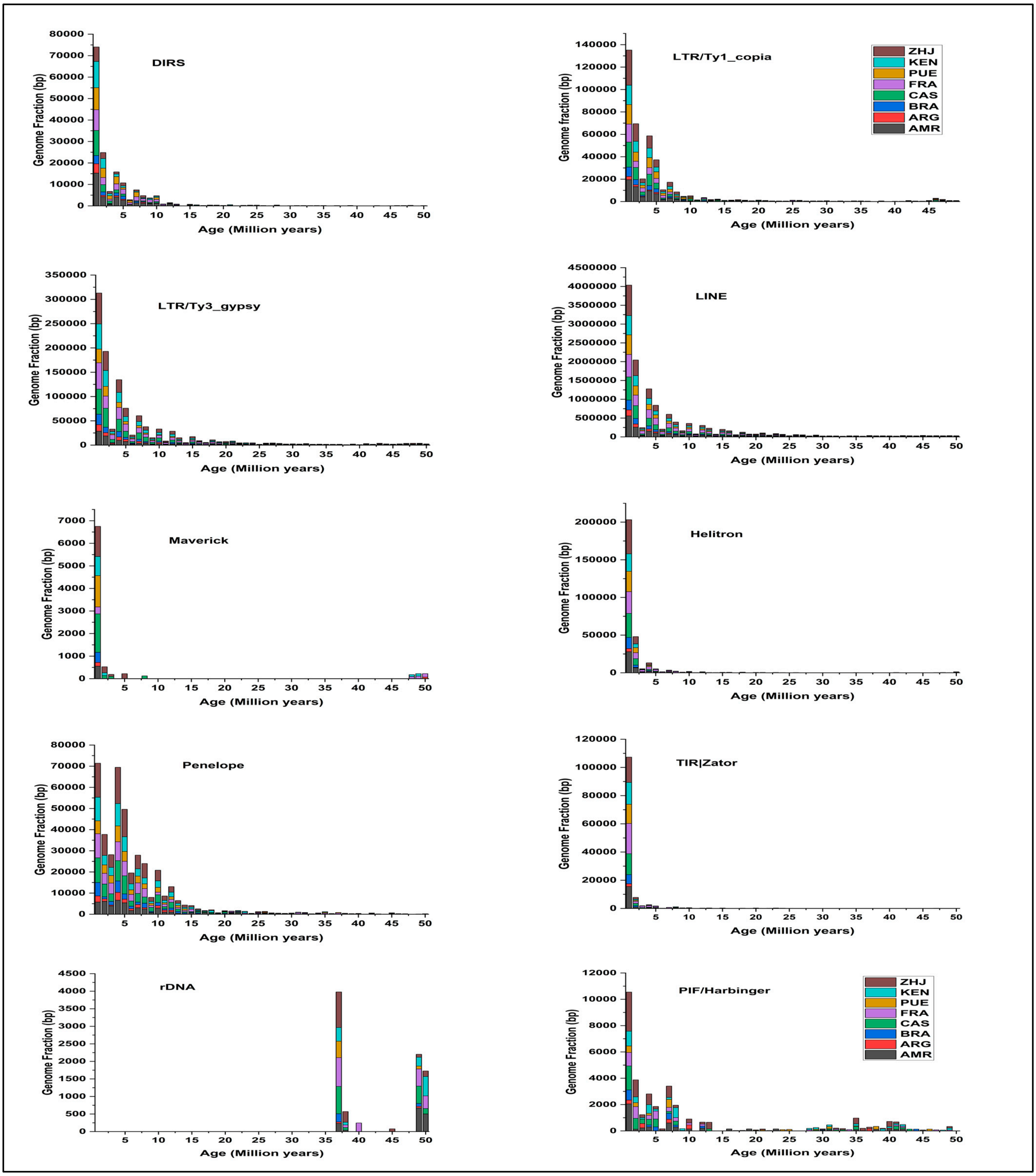

Figure S1. The interspersed repeat landscape of different transposable elements family in *Spodoptera frugiperda*. The graphs above illustrate the kimura divergence of each family from the consensus sequence, while the graph below displays the age of the components found in the genome.

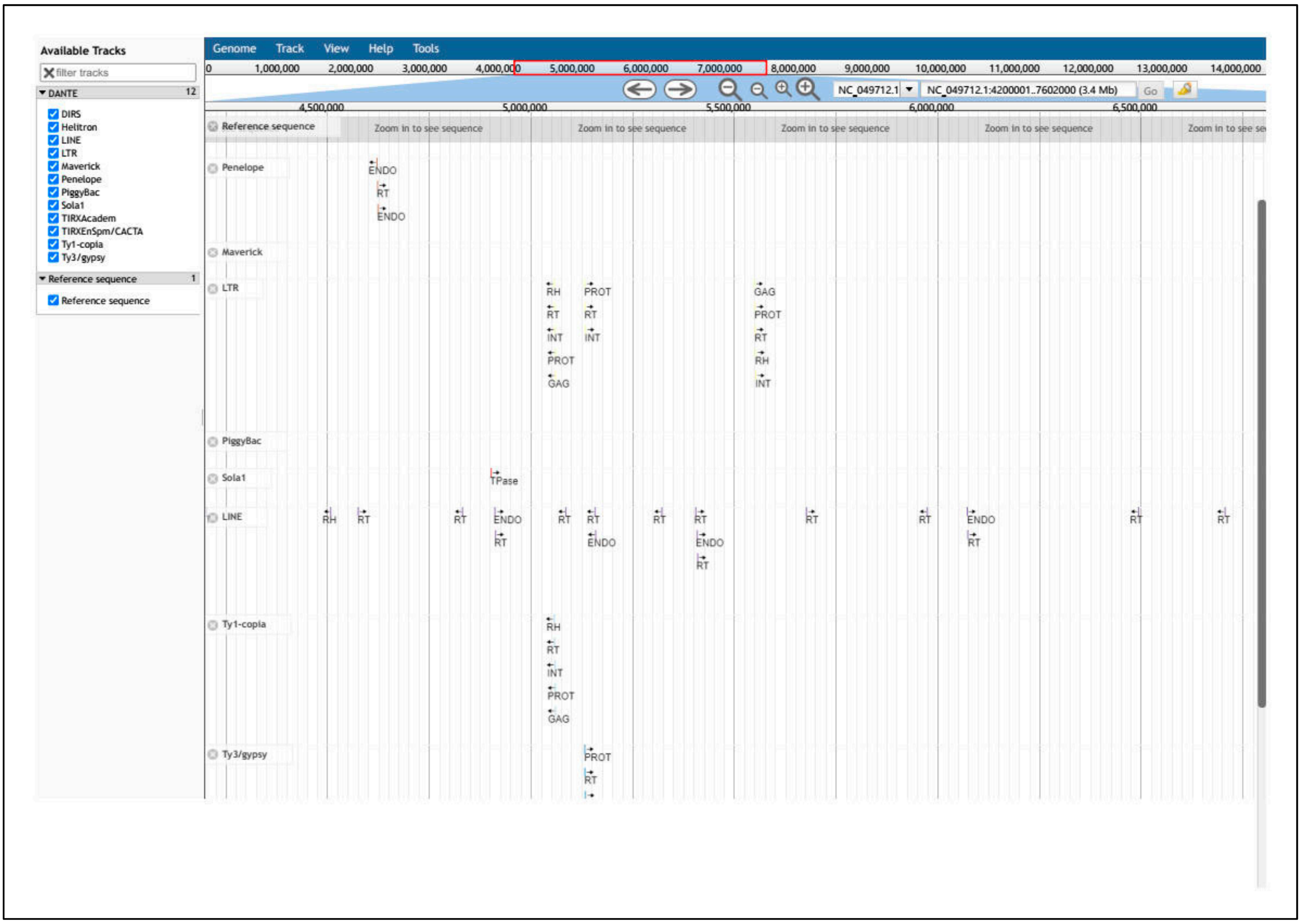

Figure S2. The genome browser output displaying the annotation tracks of different transposable elements protein domains sequences against the assembled genome of *Spodoptera frugiperda*. The assembled genome of *Spodoptera frugiperda* genome were downloaded from the NCBI and transposable elements gff3 files were extracted one by one using the DANTE tool. Then these gff3 files were uploaded as annotation track files to visualize different protein domains sequences presents in a specific TEs elements in *Spodoptera frugiperda*.

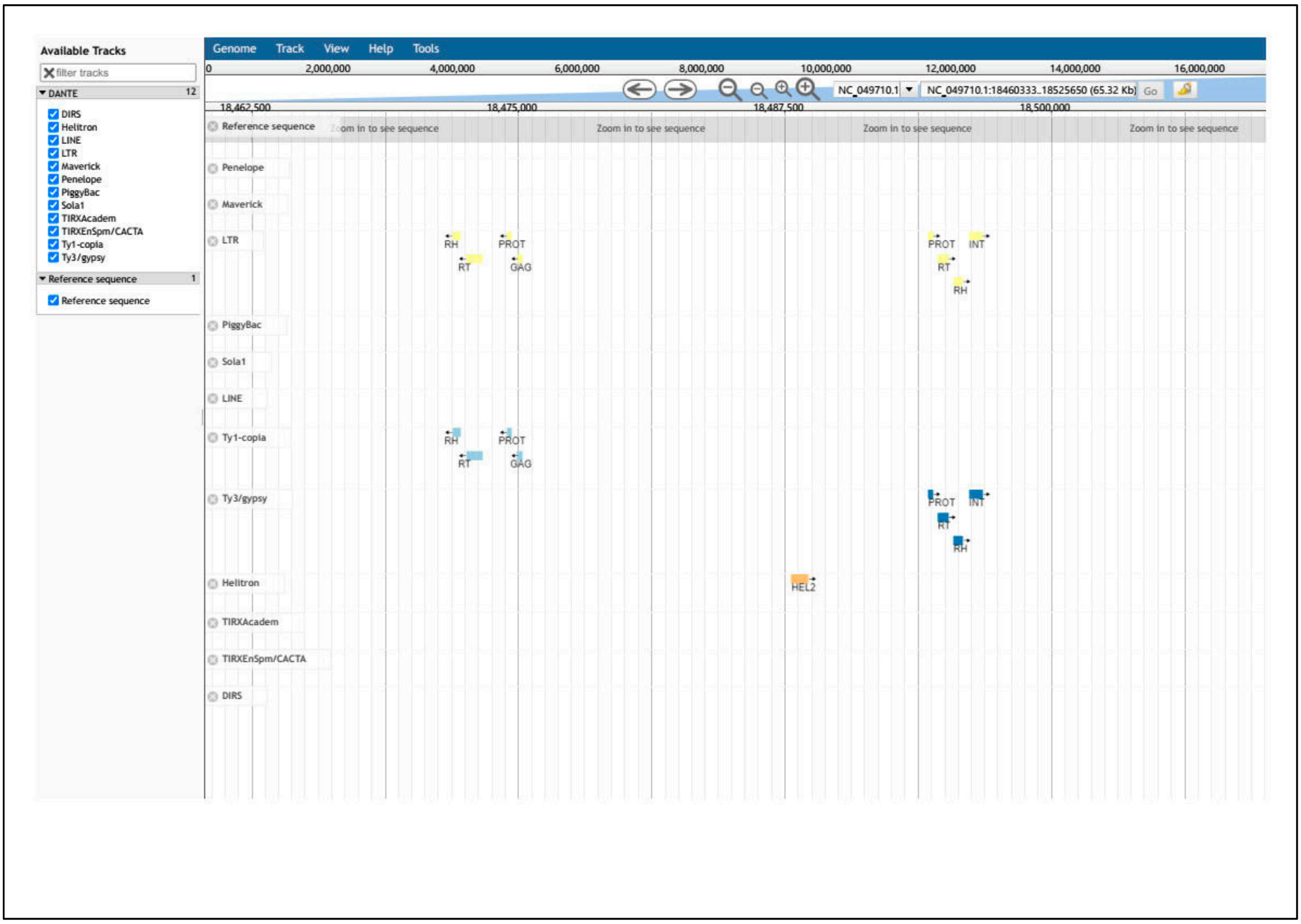

Figure S3. The genome browser output displaying the annotation tracks of LTR elements protein domains sequences against the assembled genome of *Spodoptera frugiperda*.

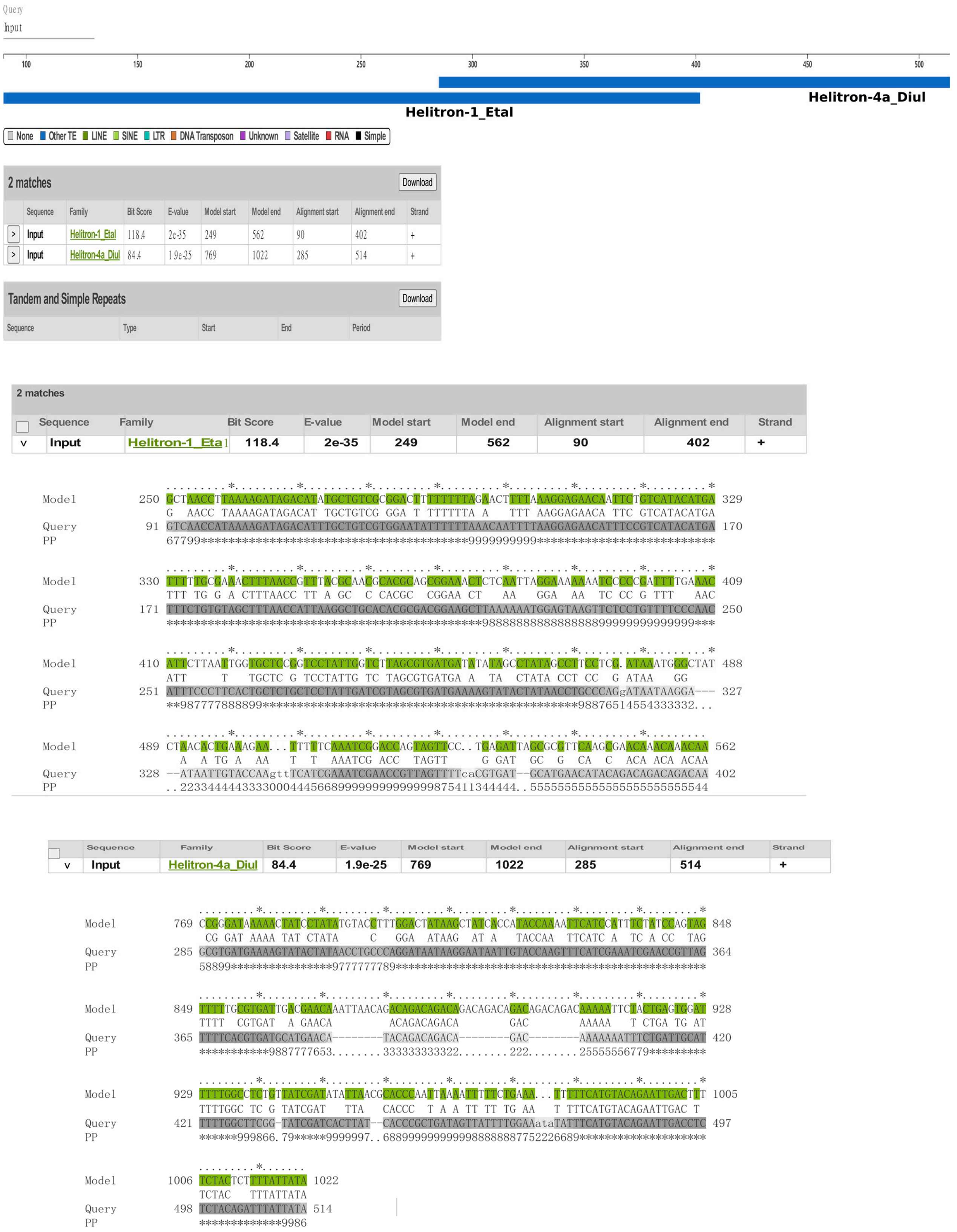

Figure S4. The SfrSat05 family revealed sequence similarity to genus Heliconius species Helitron elements (Heltron-1_Etal and Helitron-4a_Diul).

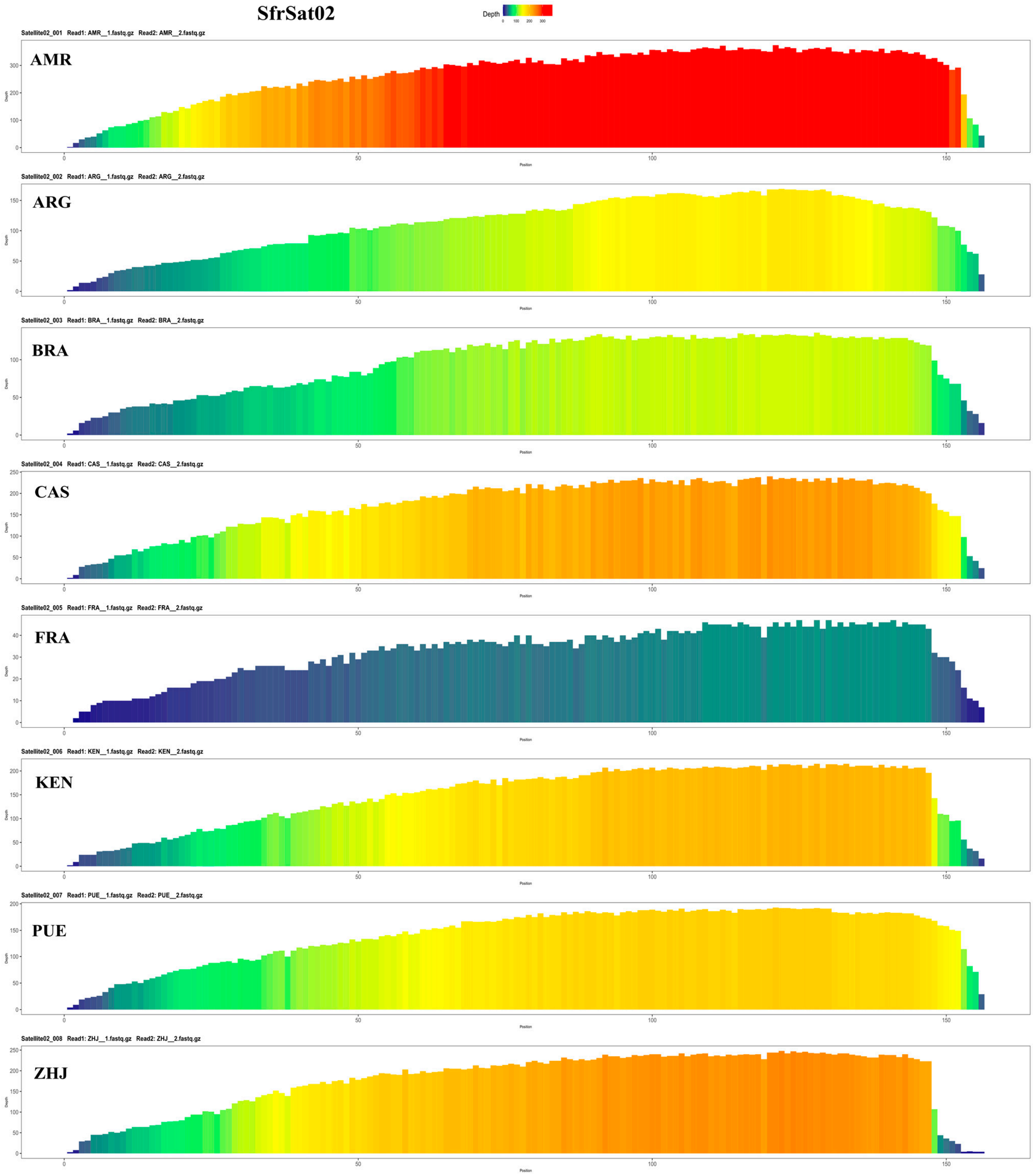

Figure S5. The colour enhanced and variant profiles of SfrSat02 satellite DNA family against eight different geographical location samples.

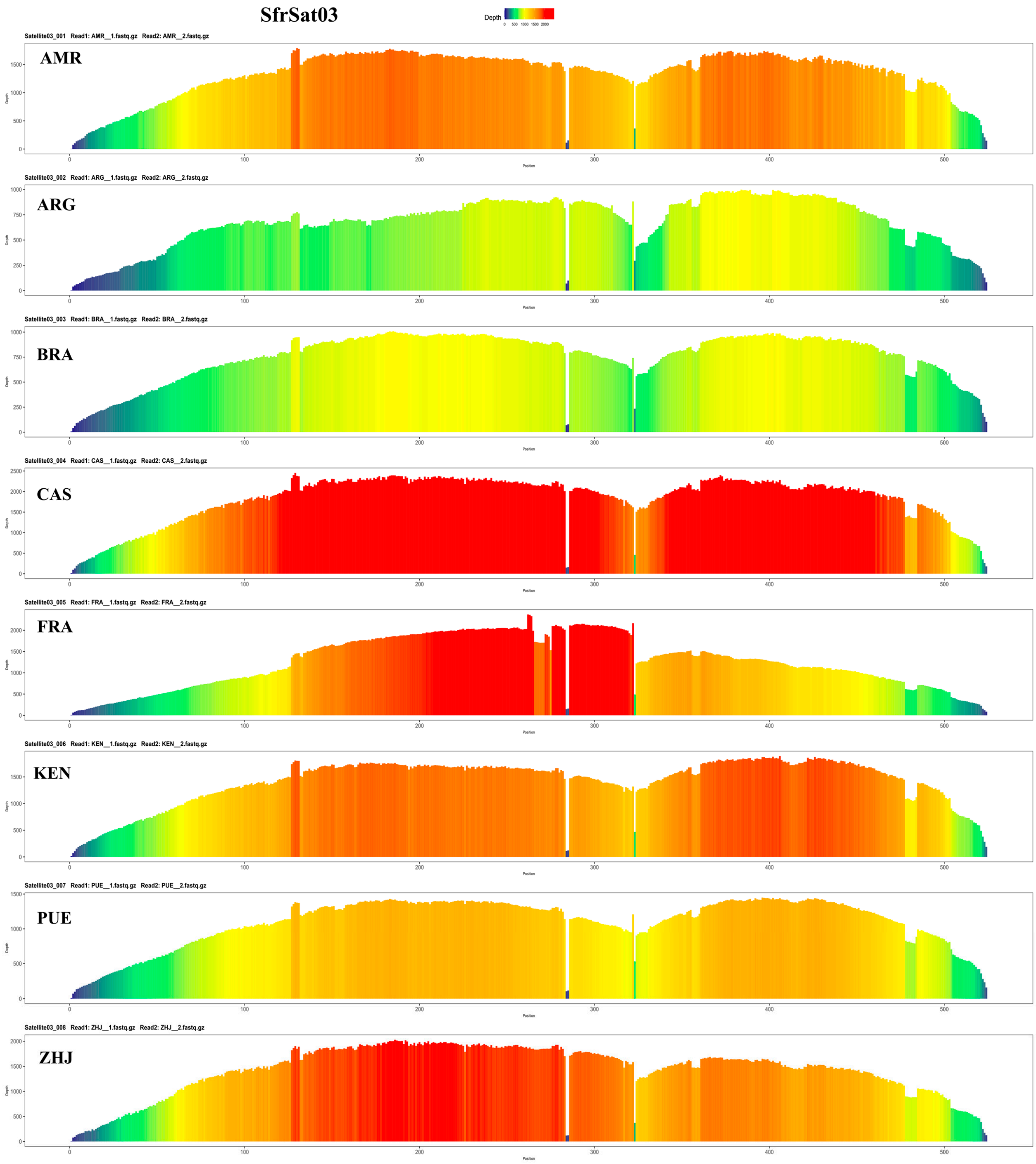

Figure S6. The colour enhanced and variant profiles of SfrSat03 satellite DNA family against eight different geographical location samples.

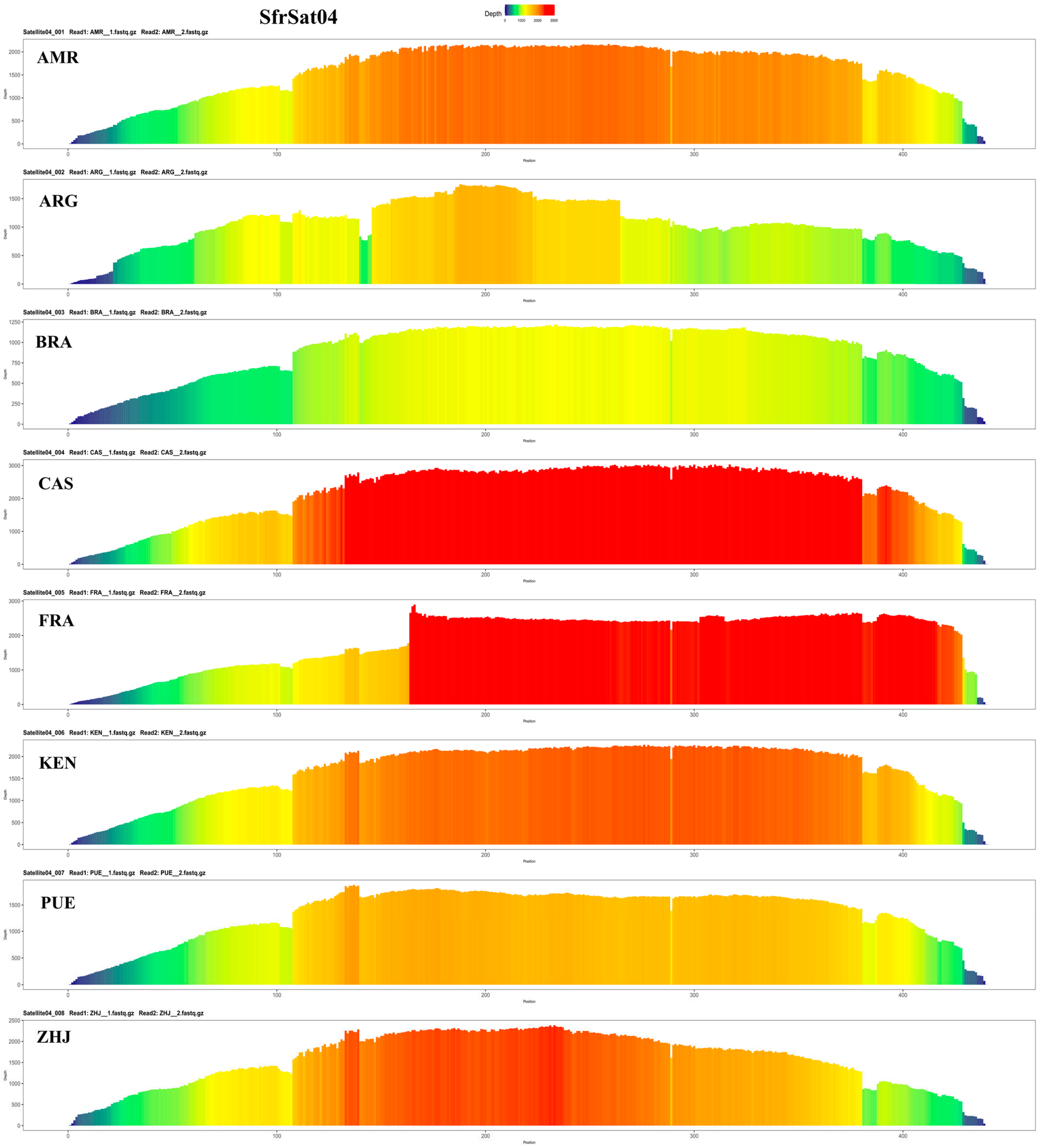

Figure S7. The colour enhanced and variant profiles of SfrSat04 satellite DNA family against eight different geographical location samples.

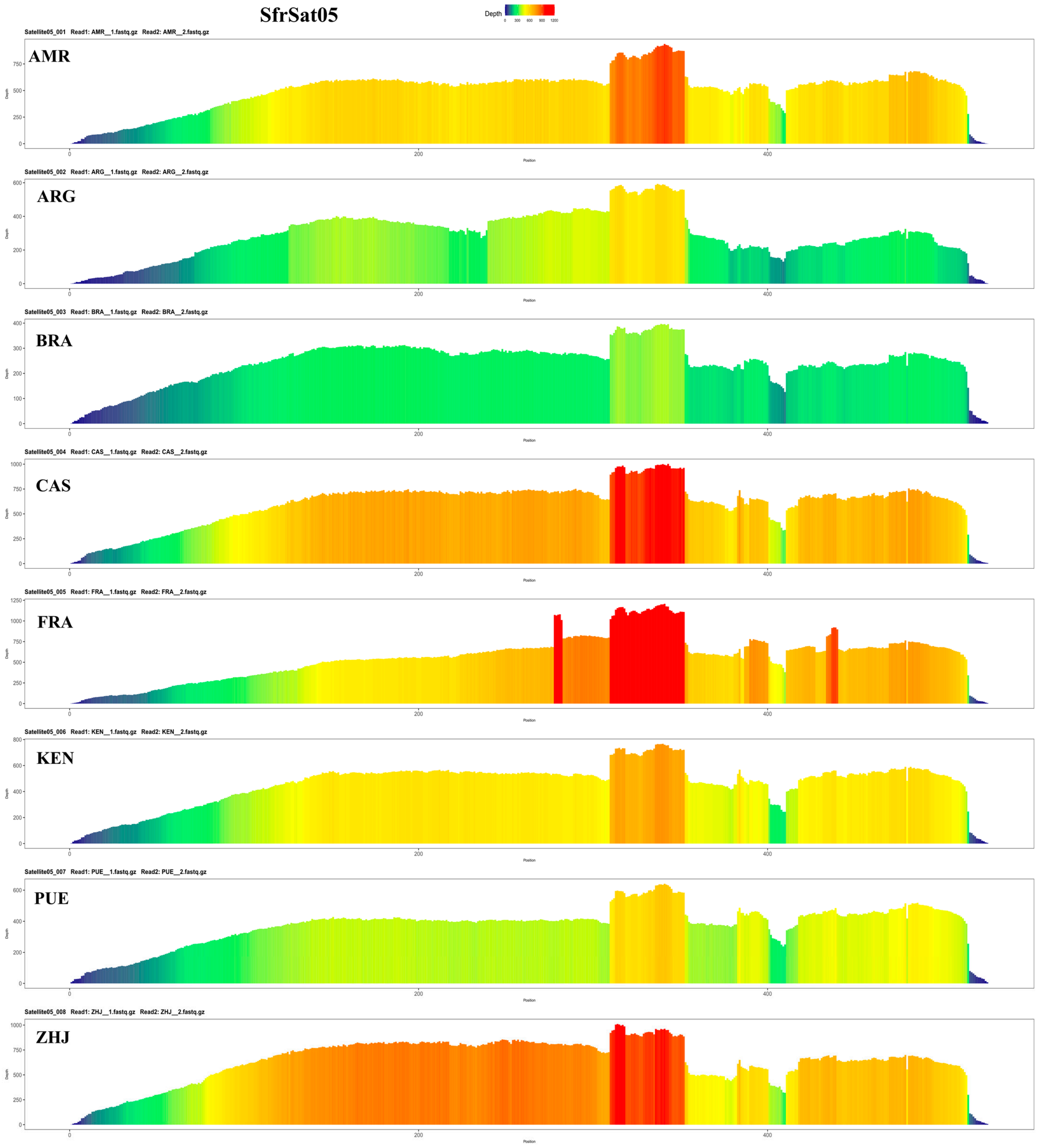

Figure S8. The colour enhanced and variant profiles of SfrSat05 satellite DNA family against eight different geographical location samples.

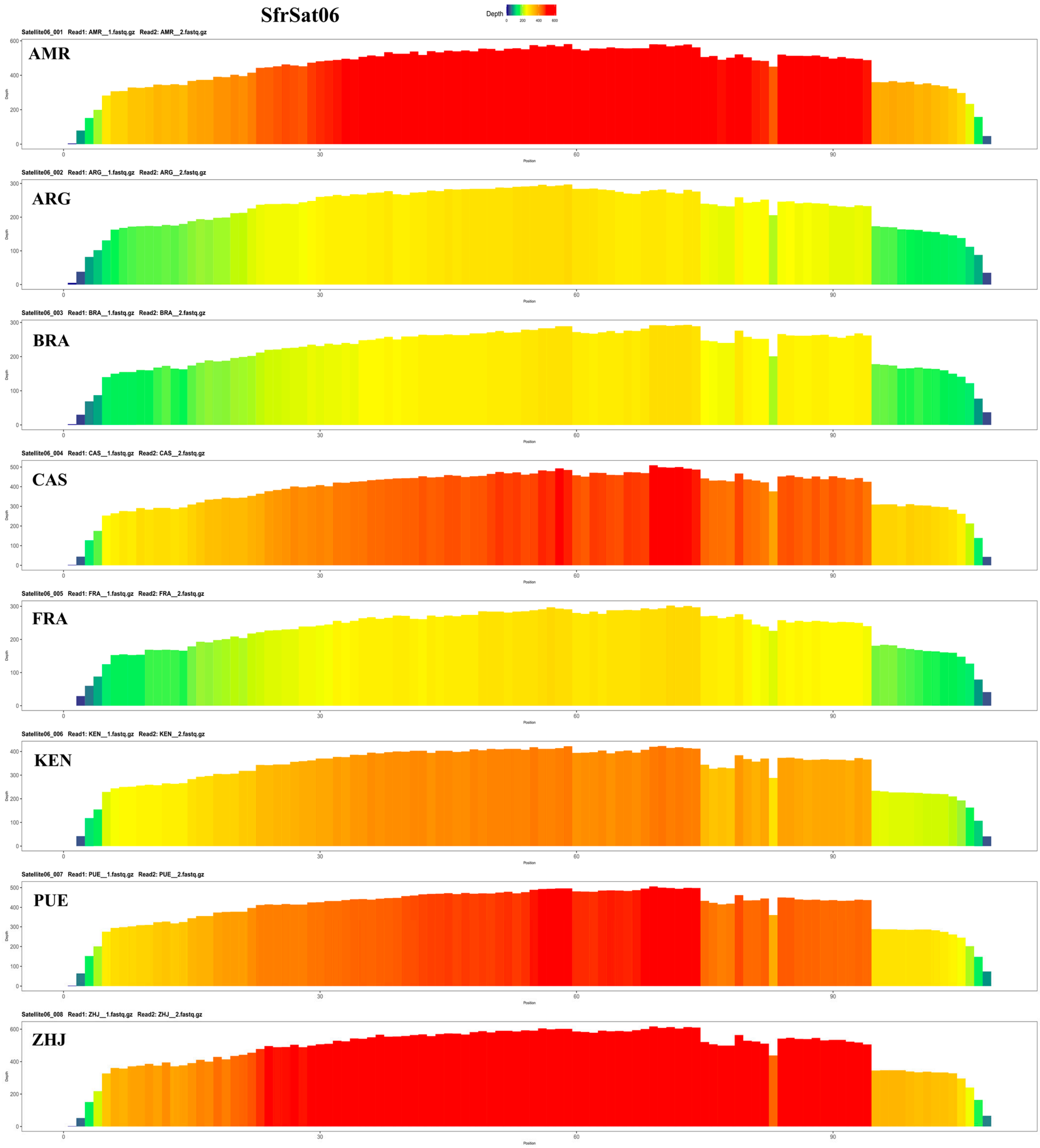

Figure S9. The colour enhanced and variant profiles of SfrSat06 satellite DNA family against eight different geographical location samples.

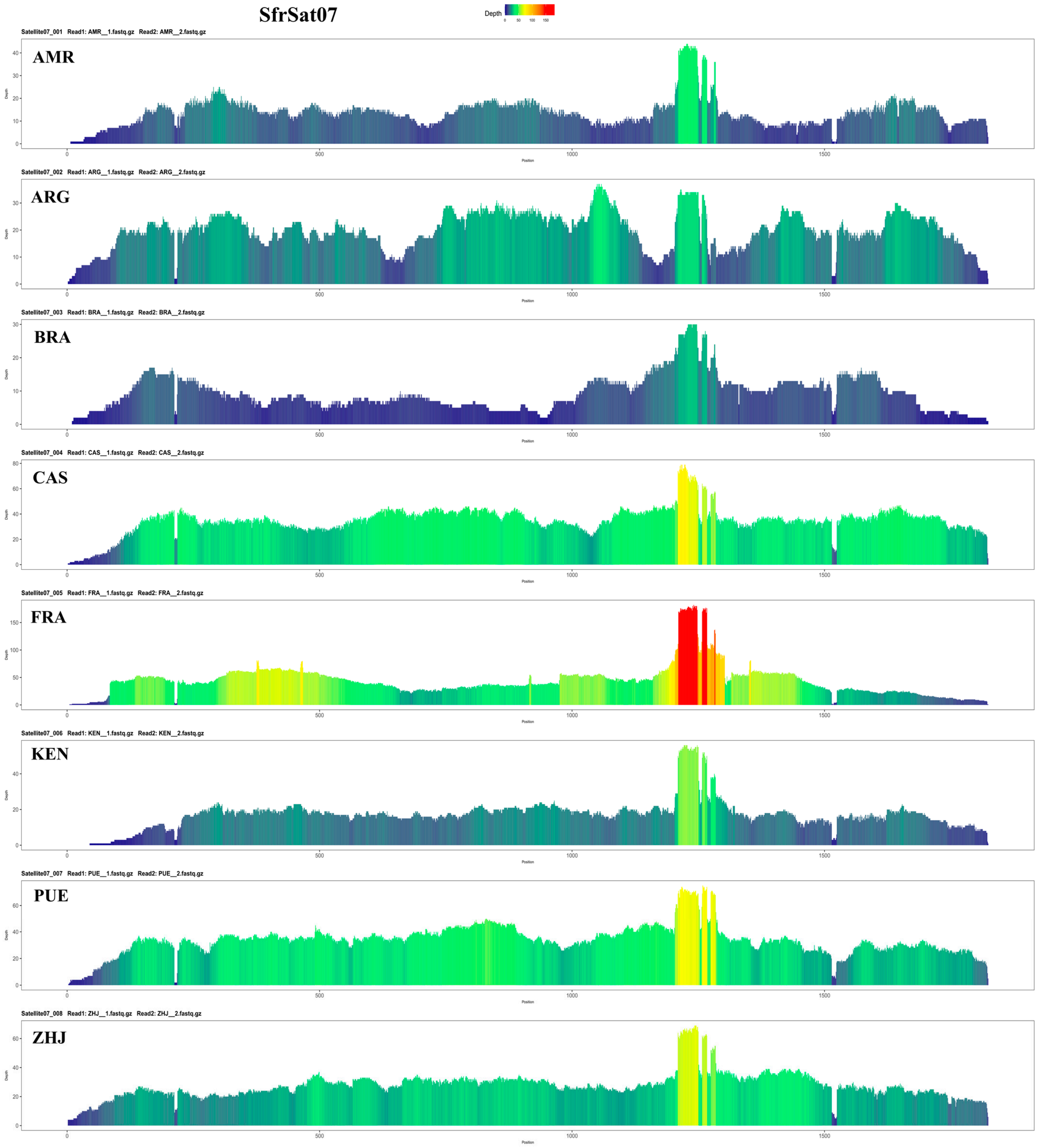

Figure S10. The colour enhanced and variant profiles of SfrSat07 satellite DNA family against eight different geographical location samples.

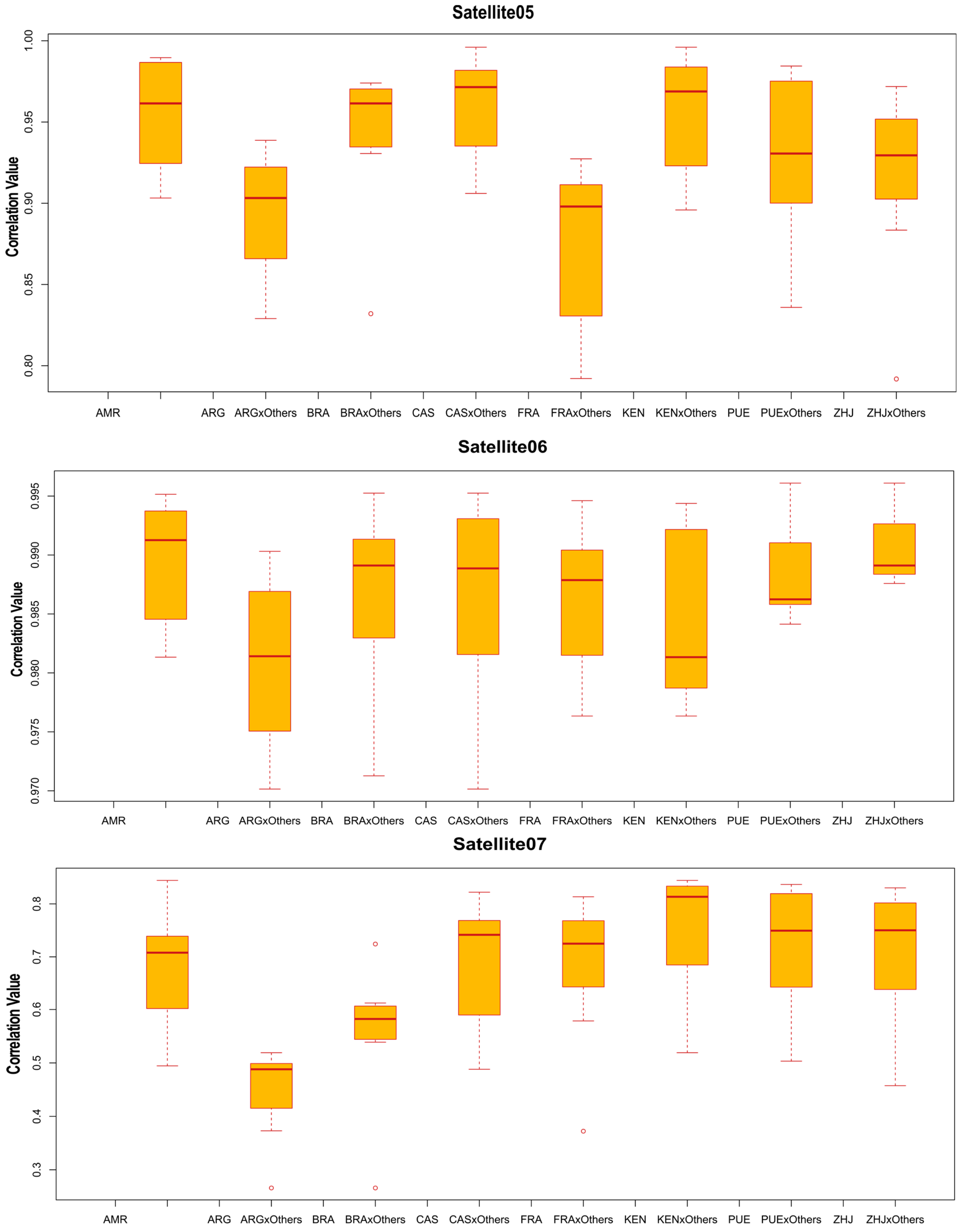

Figure S11. The correlation analysis of individual satellite DNA family (5 to 7) comparison across the three samples of each species shown similar correlation values within samples and varying in between group correlation values.

Table S1. Table contains the spearsman rank order correlation test values to infer the possible correlation between the monomer length against the A+T content, K2P divergence, percentage abundance and copy number.

	**A+T vs Length**	**Length vs Divergence**	**Length vs Abndunce**	**Length vs copy_number**	**Divergence vs Abundance**
r_s	0.18	0.5	0.14	-0.03	0.17
N	7	7	7	7	7
T-statistic	0.4	1.29	0.32	0.07	0.4
df	5	5	5	5	5
p_value	0.7	0.25	0.76	0.93	0.7
